# A Case of Non-cirrhotic Portal Hypertension With Antiphospholipid Syndrome

**DOI:** 10.7759/cureus.53843

**Published:** 2024-02-08

**Authors:** Mili Shah, Razia Gill, Priya Hotwani, Hamsika Moparty, Naresh Kumar, Dhir Gala, Vikash Kumar

**Affiliations:** 1 Internal Medicine, American University of the Caribbean School of Medicine, Sint Maarten, SXM; 2 Internal Medicine, Parkview Medical Center, Fort Wayne, USA; 3 Internal Medicine, The Brooklyn Hospital Center, Brooklyn, USA

**Keywords:** refractory ascites, transjugular intrahepatic portosystemic shunt (tips), antiphospholipid syndrome (aps), obliterative portal venopathy (opv), nodular regenerative hyperplasia (nrh), non-cirrhotic portal hypertension

## Abstract

Nodular regenerative hyperplasia (NRH) and obliterative portal venopathy (OPV) are two causes of non-cirrhotic portal hypertension (NCPH), which is a vascular liver disease wherein clinical signs of portal hypertension (PHT), such as esophageal varices, ascites, and splenomegaly develop in the absence of cirrhosis and portal vein thrombosis. The etiology often remains unidentified, but herein we present the case of a 56-year-old male with NCPH and refractory ascites who underwent liver biopsy confirming NRH and OPV. Etiological workup revealed beta-2 glycoprotein-1 and anticardiolipin antibodies, concerning antiphospholipid syndrome (APS) despite no prior history of thrombosis. The patient underwent a transjugular intrahepatic portosystemic shunt (TIPS) procedure for his refractory ascites and was started on prophylactic anticoagulation owing to a concern for APS with clinical improvement in his ascites and shortness of breath. Pursuing TIPS earlier in the setting of refractory ascites, as well as offering anticoagulation therapy for patients with possible APS to prevent the development of potential thromboses, could be appropriate recommendations to prevent complications in the disease course. This case report highlights the need for further investigations on the etiologies, diagnosis pathways, and treatment options for NCPH.

## Introduction

Non-cirrhotic portal hypertension (NCPH) is classified by increased portal pressure without evidence of cirrhosis [1]. NCPH is typically discovered in lower socioeconomic classes and developing parts of the world and can affect a broad population, although it is typically more common in males in their third and fourth decades of life [2,3]. Various theories hypothesize the pathogenesis of NCPH, and, as the exact etiology is not well understood, NCPH is a disease of exclusion. Patients suffering from NCPH often exhibit upper gastrointestinal bleeding because of esophageal varices, which also places patients at risk for secondary anemia [4]. As cirrhosis is ruled out as a cause of the increased portal pressure, further examination is required. Some patients with NCPH have been discovered to have increased vascular cell adhesion molecules (VCAM-1), connective tissue growth factor (CTGF), anti-DNA antibodies, anti-microsomal antibodies, and the presence of human leukocyte antigens (HLA)-DR3 [1]. All of which can promote fibrosis and phlebosclerosis of the portal system. The liver during NCPH can differ in its appearance with some having nodular irregularities, thickening of the liver capsule, and alteration of the portal tracts; however, even with this broad presentation, liver enzymes tend to be in range, and liver tests are often in the normal range [5,6]. Common treatments for NCPH include endoscopic variceal ligation, cyanoacrylate glue injections, and transjugular intrahepatic portosystemic shunts to decrease the risk of bleeding and varices [7]. We present a case of a 56-year-old male with NCPH and refractory ascites who underwent a liver biopsy confirming nodular regenerative hyperplasia (NRH) and obliterative portal venopathy (OPV).

This case report was previously presented as a meeting abstract at the 2022 American College of Gastroenterology Conference on October 24, 2022 (https://doi.org/10.14309/01.ajg.0000868388.08584.c6).

## Case presentation

A 56-year-old male patient, with a history of refractory ascites and NCPH, presented to the gastroenterology clinic. At presentation, his vitals were stable and within normal limits. The physical examination was significant for a distended abdomen with a positive fluid wave. Upon initial assessment, the patient was found to have NRH and OPV on liver biopsy, which was suggestive of an underlying autoimmune disorder. This prompted further investigations with a thorough etiological workup.

The patient had no prior history of thrombosis, but our investigations revealed the presence of beta-2 glycoprotein-1 and anticardiolipin antibodies, indicating a high likelihood of antiphospholipid syndrome (APS). This was an unexpected finding, as APS is usually associated with thrombotic events. Nevertheless, we started the patient on prophylactic anticoagulation therapy to reduce the risk of clot formation.

**Figure 1:**
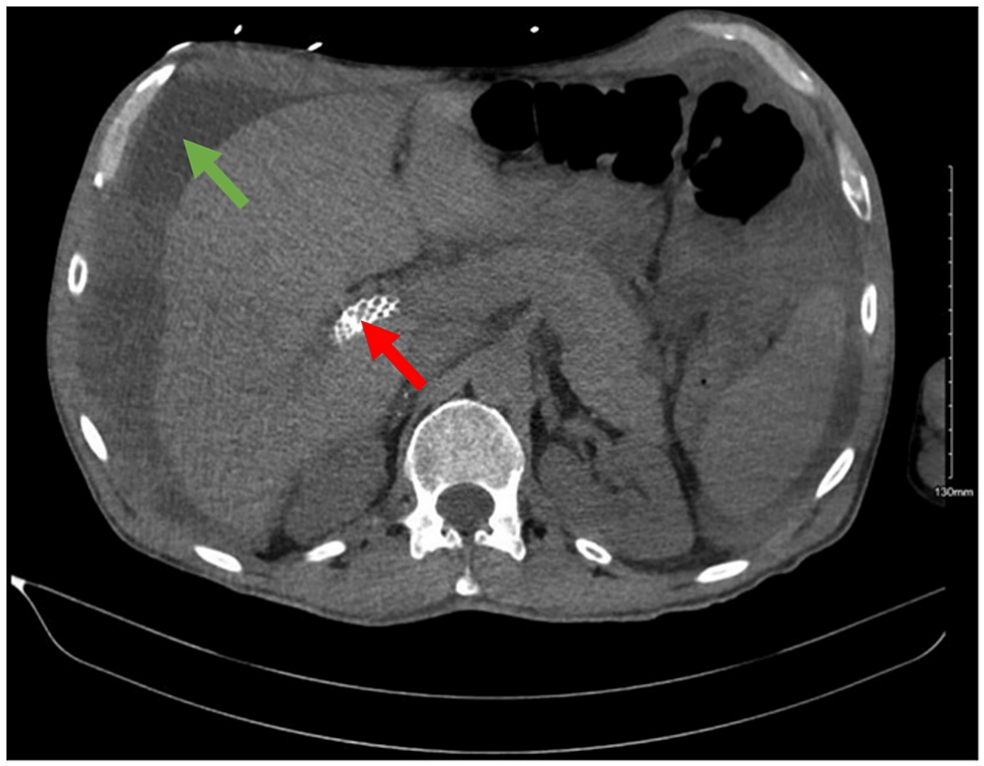
This figure depicts a CT scan of the abdomen, showing ascites (green arrow) alongside a prominently visible transjugular intrahepatic portosystemic shunt (TIPS) (red arrow).

Because of his refractory ascites, the patient underwent a transjugular intrahepatic portosystemic shunt (TIPS) procedure to alleviate his symptoms (Figure 1). The TIPS procedure involves creating a shunt between the portal hepatic veins to reduce PHT. Post-procedure, the patient was started on prophylactic anticoagulation therapy to prevent clot formation due to APS.

Following the TIPS procedure and initiation of anticoagulation therapy, the patient's clinical condition improved significantly. His ascites and shortness of breath resolved, and he was able to resume his daily activities without any difficulty. Regular follow-up visits were scheduled to monitor the patient's progress and adjust his treatment, as necessary.

Further workup revealed that the patient had a history of heavy alcohol use and a family history of liver disease. The patient denied any recent alcohol use, but his liver biopsy showed evidence of alcoholic liver disease, which was contributing to his NCPH.

In addition to starting prophylactic anticoagulation therapy and performing a TIPS procedure, the patient was advised to abstain from alcohol consumption and was started on medications to manage his ascites and liver disease. Moreover, he was referred to a nutritionist for dietary counseling and to a support group to help him quit alcohol. During follow-up visits, the patient's liver function tests and imaging studies presented gradual improvement.

## Discussion

NCPH is characterized by an increase in the portal pressure of >10 mmHg in the absence of liver cirrhosis [8]. The lesions found in NCPH are vascular and classified by the site of resistance to portal blood flow (prehepatic, hepatic, and posthepatic), and patients typically present with PHT without evidence of parenchymal dysfunction in the liver [9]. Most cases of NCPH are believed to be caused by endothelial cell lesions, intimal thickening, thrombotic obliterations, and scarring of the hepatic venous circulation along with increased blood flow [10].

Although NCPH is seen all over the world, it is more prevalent in the developing world, most notably in individuals with a lower socioeconomic class [11]. In Asia, NCPH is the most common cause of PHT, with the disease being more common in males in the third-fourth decades of life [1,3]. However, the prevalence of NCPH and different types of idiopathic PHT varies extensively between different geographic areas and has different demographics depending on the region examined. In Western populations, the median age of diagnosis with NCPH is 40 years old, while the median age of diagnosis in Asian populations tends to be younger [9]. The decreasing incidence of NCPH in countries such as Japan and the low prevalence of NCPH in Western countries, compared to developing countries, may be explained by improvements in hygiene and living standards [9].

The exact etiology of NCPH remains unknown, leaving NCPH as a diagnosis of exclusion. Several categories have been used to describe the potential pathogenesis of NCPH including immunological disorders, infections (HIV), exposure to medication or toxins, genetic disorders, and prothrombotic conditions [12,13]. Populations exposed to arsenic have been identified as a risk factor for NCPH as patients with chronic arsenic exposure demonstrated periportal fibrosis and vascular channels in portal zones as seen in NCPH [3,14]. APS is an autoimmune disorder that is characterized by thrombosis and positive antiphospholipid antibodies. APS is the fifth most common systemic risk factor for NCPH and is also known to be associated with anticardiolipin and anti-beta-2 glycoprotein antibodies [15]. 

Patients with NCPH commonly present with upper gastrointestinal bleeding due to varices, splenomegaly, and hypersplenism and typically are from a low socioeconomic background [16]. Less common presentations for patients include ascites after bleeding, jaundice, and hepatic encephalopathy [17]. Rare presentations of NCPH can also include glomerulonephritis and hypoxemia [3]. 

In NCPH, there is an increased incidence of upregulated VCAM-1, along with increased levels of CTGF, which promotes fibrosis and phlebosclerosis [1]. Patients in Japan also demonstrated anti-DNA antibodies in 69.2% of female patients, antinuclear antibodies in 24%, and anti-microsomal antibodies in 21.5% of patients supporting the immunological theory of NCPH [2]. There is also evidence that indicates that HLA class II molecules may be involved in NCPH, with HLA-DR3 making individuals more vulnerable, while HLA-DR2 may confer protection [18]. 

The appearance of the liver in NCPH is varied. In some instances, the liver may look normal, in other cases the liver may be nodular, have irregular thickening of the liver capsule, or have larger portal tracts as a result of fibrosis [5]. Another common finding in NCPH, is thrombosis of the large and small portal vein branches, along with OPV of the liver (thickening of the blood vessels), while the lobular architecture and hepatic parenchyma are unaffected [19]. Imaging results typically show a dilated and patent spleen portal axis with thickened blood vessels that have increased echogenicity. A doppler ultrasound can also be used to examine NCPH as on a doppler ultrasound, intrahepatic thrombi can be localized and identified. An endoscopy is also used to visualize if there are esophageal varices. In 85-95% of patients with NCPH, endoscopy will typically find either gastric or esophageal varices, with esophageal being more common [20].

To treat varices that commonly accompany NCPH, endoscopic variceal ligation is often used. Other options for treatment include cyanoacrylate glue injections and transjugular intrahepatic portosystemic shunts to further reduce variceal bleeding [21]. Fortunately, the prognosis of NCPH is much better than cirrhosis, with NCPH patients also having decreased mortality from acute variceal bleeding compared to cirrhotic patients. Patients who underwent endoscopic variceal eradication were also found to have a near 100% survival two and five years after treatment, while a five-year survival of 88% was found for patients who used other treatment methods such as shunt systems [3].

## Conclusions

In conclusion, NCPH is a rare disease that presents with an increase in portal pressure without evidence of cirrhosis. The pathogenesis of NCPH is still not well understood, and the disease is typically diagnosed through a process of exclusion. Patients with NCPH may experience upper gastrointestinal bleeding because of esophageal varices and are at risk for secondary anemia. Common treatments for NCPH include endoscopic variceal ligation, cyanoacrylate glue injections, and transjugular intrahepatic portosystemic shunts to decrease the risk of bleeding and varices. In this case report, we presented a 56-year-old male patient with NCPH and refractory ascites who was found to have NRH and OPV upon liver biopsy, which was suggestive of an underlying autoimmune disorder. The patient was started on prophylactic anticoagulation therapy and underwent a TIPS procedure, which resulted in significant improvement in his clinical condition. Further research is needed to better understand the pathogenesis of NCPH and identify effective treatments for this rare disease.
